# Prognostic implications of cGAS and STING gene expression in acute myeloid leukemia

**DOI:** 10.3389/ebm.2024.10108

**Published:** 2024-02-29

**Authors:** Qiuling Chen, Yan Hong, WeiFeng Chen, Feng Lin, Jiawei Zeng, Yueting Huang, Li Zhang, Jingwei Yao, Bing Xu

**Affiliations:** ^1^ The School of Clinical Medicine, Fujian Medical University, Fuzhou, Fujian, China; ^2^ Department of Hematology, The First Affiliated Hospital of Xiamen University and Institute of Hematology, School of Medicine, Xiamen University, Xiamen, China; ^3^ Department of Hematology, Shantou Central Hospital, Shantou, Guangdong, China

**Keywords:** cGAS, STING, expression, prognosis, acute myeloid leukemia

## Abstract

Acute myeloid leukemia (AML) is one of the most threatening hematological malignances. cGAS-STING pathway plays an important role in tumor immunity and development. However, the prognostic role of cGAS-STING pathway in AML remains unknown. Firstly, The expression of cGAS and STING was analyzed by bioinformatics analysis. Subsequently, Bone marrow samples were collected from 120 AML patients and 15 healthy individuals in an independent cohort. The cGAS and STING expression was significantly elevated in AML patients compared with healthy controls. Patients with high cGAS and STING expression had a higher NRAS/KRAS mutation rate and lower complete remission (CR) rate. High cGAS and STING expression was significantly associated with lower overall survival (OS) and disease-free survival (DFS). Our findings revealed that the expression levels of cGAS and STING in AML are elevated. High expression of cGAS and STING correlated with worse OS and DFS and may be a useful biomarker for inferior prognosis in AML patients.

## Impact statement

Acute myeloid leukemia (AML) is an aggressive hematopoietic malignancy with a high incidence rate and poor clinical prognosis. However, the current understanding of the molecular mechanism of AML development and progress is very limited. In our study, we evaluated the expression of cGAS and STING by collecting bone marrow samples from 120 AML patients and 15 healthy individuals, and found that cGAS-STING pathway was involved in the pathogenesis of AML. The high expression of cGAS and STING is related to the worse OS and DFS, which may be useful biomarkers for the poor prognosis of AML patients. Our research fills the gap in the pathogenesis of AML and provides potential biomarkers for clinical diagnosis and treatment.

## Introduction

Acute myeloid leukemia (AML) is an aggressive hematopoietic malignant disease resulted in high morbidity and unfavorable clinical outcome [1]. Cytogenetics is the backbone for risk stratification, facilitating the classification of AML patients into favorable, intermediate, and poor prognostic groups. However, more than half of AML patients are classified as an intermediate cytogenetic risk group but their clinical outcomes turn out to be distinct [2, 3]. A large percentage of AML patients will suffer from disease recurrences due to the heterogeneous AML clones [4, 5]. The current perception of molecular mechanisms on the development and progression of AML is limited to date [6]. Thus, the identification of new molecular biomarker and revealing of novel mechanism are needed to prompt a more precise risk stratification and develop targeted therapies for AML.

As a vital DNA sensor, cyclic guanosine monophos-phate (GMP)-adenosine monophosphate (AMP) (cGAMP) synthase (cGAS) initiated an innate immunity pathway through binding deviant DNA in the cytosol. cGAMP can activate stimulator of interferon genes (STING), leading to a signaling cascade which produces type I interferons and other functional cytokines [7]. cGAS-STING pathway was previously introduced as a crucial initiator of innate immune and anti-virus responses [8]. Recent studies have revealed the multiple role of cGAS-STING pathway in cancer. Activated cGAS-STING pathway in tumor cells lead to upregulation of various inflammatory genes, such as Type I interferon and impedes the neoplastic progression [9]. There are also related reports in AML, and therefore, many scientists have attempted to increase the expression of type I interferons by upregulating STING, thus achieving the goal of treating AML [10–13]. Yet, mounting evidence indicates that cGAS-STING pathway might provoke inflammation, leading to tumor transformation, development and metastasis in certain diseases [14–16]. Consequently, the relationship between the expression levels of cGAS and Sting in AML and patient prognosis remains unclear.

In this study, we found that cGAS and STING expression levels were higher in AML patients compared with healthy controls by using Gene Expression Profiling Interactive Analysis (GEPIA) and Gene-Set Enrichment Analysis (GSEA) with further validation performed in our cohort. Furthermore, we investigated the impact of cGAS and STING expression on the clinical outcomes of AML patients. Our results indicated that higher expression of cGAS and STING was associated with inferior survival in AML patients.

## Materials and Methods

### Datasets

The GEPIA^1^ integrated the two databases including The Cancer Genome Atlas (TCGA)^2^ and the Genotype-Tissue Expression Project (GTEx)^3^. This platform can perform gene expression analysis based on RNA-seq expression data for 9,736 tumor samples and 8,587 control samples [17]. In this study, the GEPIA was used to analyze the expression of cGAS and STING in different tumors. Two gene expression profile datasets (GSE63270 and GSE30029) which involved expression data for healthy and AML bone marrow samples were downloaded from Gene Expression Omnibus (GEO,^4^) [18].

### Clinical patients and reverse transcribed quantitative PCR (RT-qPCR)

A total of 120 non-M3 AML patients diagnosed in our department during 2018–2020 were enrolled in the present study. Additionally, 15 healthy allo-HSCT donors were enrolled as control. All patients were diagnosed and classified according to French -American—British (FAB) group and World Health Organization (WHO) classification. Complete remission (CR) was defined by <5% blast cells in the bone marrow and normalization of the peripheral blood counts at 4 weeks after starting induction therapy, without any evidence of extramedullary disease. The written informed consents were provided from all patients in accordance with Declaration of Helsinki.

Ficoll-Hypaque density gradient column (Cytova, Uppsala, Sweden) was used to isolate monocytes. The total RNA was isolated from monocytes by Trizol (Invitrogen, United States), then reverse transcribed into cDNA using BioTeke super RT Kit (BioTeke, Beijing, China). RT-qPCR was performed with an ABI PRISM 7500 real-time PCR system (PE Applied Biosystems, Foster City, CA, United States). We selected β-actin as a control gene to compensate for variations in quality and quantity of RNA and cDNA. The amplification conditions were as follows: 95 °C for 2 min, followed by 40 cycles of 95 °C for 15 s and 60 °C for 30 s. Sequences were as follows:

cGAS - Forward: 5′- CAC​GAA​GCC​AAG​ACC​TCC​G -3′

cGAS - Reverse: 5′- GTC​GCA​CTT​CAG​TCT​GAG​CA -3′

STING - Forward: 5′- CCA​GAG​CAC​ACT​CTC​CGG​TA -3′

STING - Reverse: 5′- CGC​ATT​TGG​GAG​GGA​GTA​GTA -3′

β-actin - Forward: 5′- TGT​GGC​ATC​CAC​GAA​ACT​AC -3′

β-actin - Reverse: 5′- GGA​GCA​ATG​ATC​TTG​ATC​TTC​A -3′

The relative expression levels of above genes were calculated using the 2^−ΔΔCT^ method (fold change over control expression).

### Statistical analysis

All data were analyzed with SPSS (version 20.0; Chicago, IL) and GraphPad Prism 5.0 (GraphPad Software Inc., United States). Overall survival (OS) was calculated from the date of diagnosis until death caused by any reason. Disease-free survival (DFS) was defined as the time from achievement of CR to relapse or the last follow-up. Mann–Whitney *U* test and Chi-square test were used for continuous and categorical variables respectively. The probabilities of OS and DFS were estimated using the Kaplan-Meier method. The expression of cGAS and STING as well as other variables were included in the univariate analysis. Only variables with *p* < 0.1 were included in a Cox proportional hazards model with time-dependent variables. Spear-man rank correlation was used to analyze the correlation between two variables. Unless otherwise specified, *p* values were based on two-sided hypothesis tests. Alpha was set at 0.05.

## Results

### Elevated expression levels of cGAS and STING in AML

**FIGURE 1:**
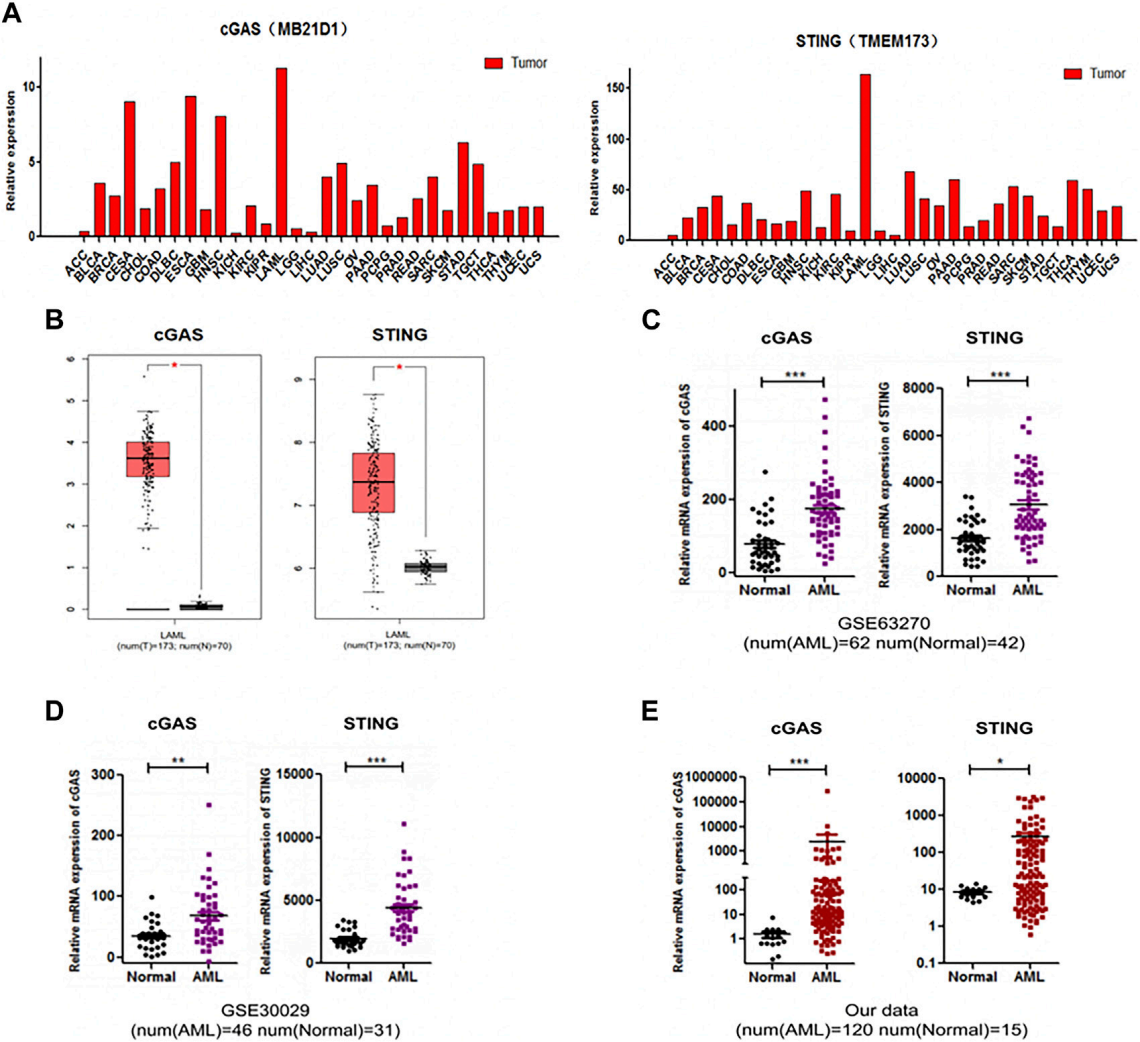
The expression levels of cGAS (MB21D1) and SING (TMEM173) were elevated in AML. **(A)** Analysis of the expression of cGAS and SING in different types of tumors, from GEPIA database. **(B–D)** Up-regulated cGAS and STING expression in AML patients compared with normal controls, from GEPIA database (173 AML and 70 normal controls), GEO: GSE63270 (62 AML and 42 normal controls) and GSE30029 (46 AML and 31 normal controls), respectively. **(E)** Validation of cGAS and STING expression of AML patients and normal controls in an independent cohort (120 AML and 15 normal controls). *p < 0.05; **p < 0.01; ***p < 0.001.

Firstly, we analyzed the expression levels of cGAS and STING in different types of tumors using GEPIA database. The analysis revealed that cGAS and STING expression levels were higher in AML compared to other tumors (Figure 1A). In addition, the dataset from GEPIA which involved 173 AML and 70 healthy control samples showed that cGAS and STING gene expression was higher in AML samples than healthy controls (Figure 1B). We then analyzed available molecular data from GSE63270 (involved 62 AML and 42 normal controls) and GSE30029 (involved 46 AML and 31 normal controls). In both cohorts, cGAS and STING gene expression levels were higher in AML samples than normal controls in consistence with the result from GEPIA analysis (*p* < 0.0001, Figures 1C, D). To validate the results from above publicly available datasets, we collected bone marrow samples from 120 AML patients and 15 healthy donors and performed the detection of cGAS and STING gene expression. We found that the expression of cGAS and STING was also elevated in AML patients from our cohort (*p* < 0.0001, Figure 1E). Taken together, these results demonstrate that up-regulation of cGAS and STING is a common feature in AML.

### Patient characteristics

The baseline characteristics of patients are shown in Table 1. We dichotomized the patients into two high and low groups based on the median values of cGAS and STING expression respectively. The distribution of risk stratification was significantly different in cGAS high and low groups with more patients at high risk in cGAS high group (*p* = 0.040). Patients with higher cGAS expression had a higher NRAS/KRAS mutation rate (*p* = 0.040) and lower CR rate (*p* < 0.0001). Similarly, patients in STING high group had a higher NRAS/KRAS mutation rate (*p* = 0.002) and tended to have a lower CR rate (*p* = 0.076). The other characteristics including age, white blood count, hemoglobin, platelet, lactate dehydrogenase, bone marrow blast percentage, karyotypes, FLT3-ITD mutation, isolated biallelic CEBPA mutation, NPM1 mutation and other mutation between patients with high and low cGAS or STING expression were not significantly different.

**TABLE 1:** Clinical characteristics of AML patients.

Variable	Total (*n* = 120)	cGAS expression levels			STING expression levels		
		Low (*n* = 60)	High (*n* = 60)	*p*-value	Low (*n* = 60)	High (*n* = 60)	*p*-value
Sex, male/female	70/50	31/29	38/22	0.268^a^	36/24	33/27	0.712^a^
Median age, year (range)	52 (3–91)	53 (3–86)	49 (5–91)	0.992^b^	52 (3–86)	51 (5–91)	0.940^b^
Median WBC, ×10^9^/L (range)	18.81 (0.25–279.03)	17.26 (0.25–241.00)	20.05 (1.10–279.03)	0.735^b^	11.58 (0.25–241.00)	22.87 (1.10–279.03)	0.062^b^
Median Hemoglobin, g/L (range)	72 (21–130)	70 (21.0–130)	73 (33–130)	0.729^b^	69.5 (21.0–130.0)	73 (29–130)	0.555^b^
Median Platelet, ×10^9^/L (range)	41 (3–487)	43 (3–487)	35 (5–157)	0.425^b^	44 (3–487)	35 (5–159)	0.910^b^
Median LDH, U/L (range)	431 (94–3,261)	436 (94–2,450)	390 (154–3,261)	0.410^b^	394 (94–2,450)	433 (150–3,261)	0.315^b^
BM blast, % (range)	59.2 (5.5–96.5)	60.8 (13.0–96.5)	51.2 (5.5–95.5)	0.371^b^	59.9 (20.5–96.5)	54.0 (5.5–95.5)	0.579^b^
Karyotype				0.268^a^			0.857^a^
Normal	55 (45.8%)	29 (48.3%)	26 (43.3%)		28 (46.7%)	27 (45.0%)	
Complex	12 (10.0%)	5 (8.3%)	7 (11.7%)		6 (10%)	6 (10%)	
t (8; 21) or inv (16) or t (16; 16)	17 (14.2%)	7 (11.7%)	10 (16.7%)		10 (16.7%)	7 (11.7%)	
Others	25 (20.8%)	16 (26.7%)	9 (15.0%)		12 (20.0%)	13 (21.7%)	
Missing	11 (9.2%)	3 (5.0%)	8 (13.3%)		4 (6.7%)	7 (11.7%)	
Risk Stratification				**0.040** ^a^			0.138^a^
Low	21 (17.5%)	15 (25.0%)	6 (10.0%)		15 (25%)	6 (10%)	
Moderate	25 (20.8%)	15 (25.0%)	10 (16.7%)		13 (21.7%)	12 (20.0%)	
High	63 (53.3%)	27 (45.0%)	36 (61.7%)		28 (46.7%)	35 (58.3%)	
Missing	11 (9.2%)	3 (5.0%)	8 (13.3%)		4 (6.7%)	7 (11.7%)	
FLT-ITD mutation (+/−)	22/86	12/44	10/42	0.815^a^	10/45	12/41	0.637^a^
Isolated biallelic CEBPA mutation (+/−)	16/92	10/46	6/46	0.423^a^	8/47	8/45	1^a^
NPM1 mutation (+/−)	13/95	8/48	5/47	0.560^a^	7/48	6/47	1^a^
AML-ETO (+/−)	11/97	3/53	8/44	0.115^a^	6/49	5/48	1^a^
ASXL1 mutation (+/−)	21/87	10/46	11/41	0.807^a^	13/46	8/41	0.333^a^
RUNX1 mutation (+/−)	16/92	7/49	9/43	0.591^a^	8/47	8/45	1
IDH1 or IDH2 mutation (+/−)	25/83	13/43	12/40	1.000^a^	13/42	12/41	0.237^a^
DNMT3A mutation (+/−)	14/94	7/49	7/45	1.000^a^	6/49	8/45	0.576^a^
TET2 mutation (+/−)	18/90	10/46	8/44	0.800^a^	8/47	10/43	0.612^a^
NRAS or KRAS mutation (+/−)	17/91	4/51	13/40	**0.040** ^a^	2/53	15/38	**0.002** ^a^
CBFB-MYH11 (+/−)	5/103	4/52	1/51	0.365^a^	3/52	2/51	1^a^
CR (+/−)	69/28	43/6	26/22	**<0.0001** ^a^	39/10	30/18	0.076^a^

Abbreviation: WBC, white blood cell; LDH, lactate dehydrogenase; BM, bone marrow; CR, complete remission.

^a^ χ2 test.

^b^ Mann-Whitney *U* test.

Bold values represent that *p* ≤ 0.05.

### High cGAS and STING expression correlated to inferior survival in AML

**FIGURE 2:**
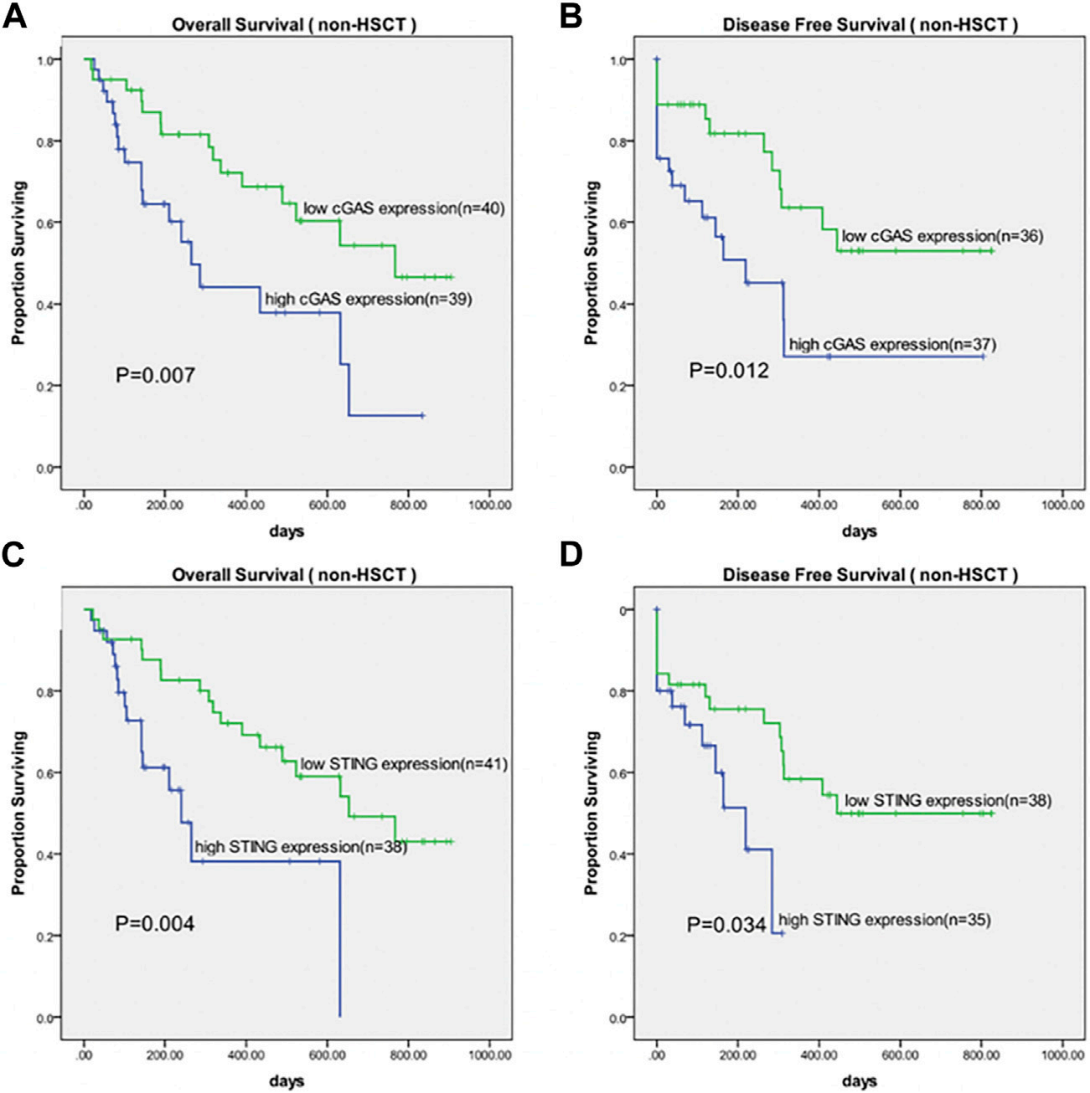
Kaplan-Meier survival curves of OS and DFS in patients grouped by median values of cGAS and STING expression. **(A)** Survival curves of OS in cGAS high and low groups. **(B)** Survival curves of DFS in cGAS high and low groups. **(C)** Survival curves of OS in STING high and low groups. **(D)** Survival curves of DFS in STING high and low groups. OS, overall survival; DFS, disease-free survival.

We further analyzed the overall survival (OS) in an adjusted cohort which excluded 18 untreated patients, 18 patients received allo-HSCT and 5 patients lost to follow-up. The remaining patients received similar treatments. In analysis of disease-free survival (DFS), 6 patients who didn’t achieve CR after treatment were further excluded. Kaplan-Meier analysis showed that patients with higher cGAS expression had a shorter OS (377.6 vs. 626.7 days, *p* = 0.007, Figure 2A) as well as a shorter DFS (312.2 vs. 543.9 days, *p* = 0.012, Figure 2B). Likewise, patients with higher STING expression had a shorter OS (332.9 vs. 612.7 days, *p* = 0.004, Figure 2C) as well as a shorter DFS (178.3 vs. 507.8 days, *p* = 0.034, Figure 2D).

### Univariate and multivariate analysis of factors affecting OS and DFS

The correlation between high cGAS and STING expression with inferior survival in AML patients indicated cGAS and STING expression levels might be of prognostic importance for AML. The results of univariate and multivariate analysis of factors affecting OS and DFS were shown in Table 2. The factors shown in patient characteristics were included in univariate analysis and variables with *p* < 0.1 were further analyzed in multivariate analysis. In multivariate Cox regression analysis, high cGAS expression was associated with inferior OS (HR = 2.348, 95% CI: 1.012–5.447; *p* = 0.047) and DFS (HR = 2.420, 95% CI: 1.071–5.469; *p* = 0.034). Nevertheless, it showed that there was no association between STING gene expression and OS or DFS. Besides, DNMT3a mutation, TET2 mutation were shown to be associated with worse OS and DFS while receiving allo-HSCT was associated with improved OS and DFS.

**TABLE 2:** Multivariate analysis of factors associated with OS and DFS.

Variable	Univariate analysis		Multivariate analysis	
	HR (95% CI)	*p*-value	HR (95% CI)	*p*-value
OS				
cGAS expression	2.910 (1.378–6.146)	0.005	2.348 (1.012–5.447)	0.047
STING expression	2.855 (1.282–6.358)	0.01		
DNMT3a mutation	2.543 (1.008–6.418)	0.048	3.420 (1.254–9.324)	0.016
TET2 mutation	2.039 (0.936–4.446)	0.073	2.521 (1.050–6.048)	0.038
allo-HSCT	0.205 (0.049–0.862)	0.031	0.171 (0.040–0.737)	0.018
DFS				
cGAS expression	2.411 (1.119–5.195)	0.025	2.420 (1.071–5.469)	0.034
DNMT3a mutation	2.568 (1.015–6.5)	0.047	3.900 (1.441–10.774)	0.008
TET2 mutation	2.481 (1.090–5.645)	0.03	2.936 (1.189–7.250)	0.02
allo-HSCT	0.198 (0.047–0.836)	0.028	0.158 (0.035–0.709)	0.016

### Positive correlation between cGAS and STING gene expression

**FIGURE 3:**
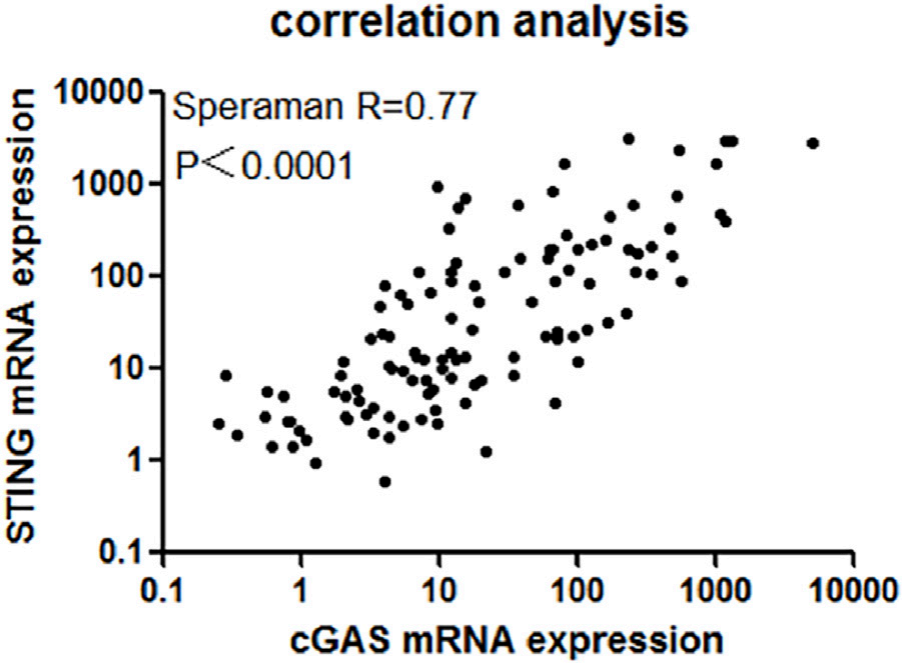
There existed a strong positive correlation between cGAS and STING expression (*R* = 0.77, 95% CI: 0.6884–0.8301, *p* < 0.0001).

As the above results showed, cGAS and STING expression was up-regulated in AML patients compared with normal controls. Although cGAS and SING are the entry of cGAS-STING pathway, previous study showed cGAS and STING expression could be regulated inconsistently in NSCLC [19]. We wondered whether cGAS and STING were up-regulated in a synchronized manner on the context of AML. Our result showed that in AML, cGAS expression was positively correlated with STING expression (R = 0.77, 95% CI: 0.6884–0.8301, *p* < 0.0001, Figure 3).

## Discussion

AML is a highly genetically heterogeneous malignant myeloproliferative disorder of bone marrow, accounting for ∼10% of all hematological diseases [1, 20]. While the next-generation sequencing technology has been tremendously developed, numerous recurrent point mutations, epigenetic changes as well as cytogenetic abnormality have been thoroughly recognized [21, 22]. Cytogenetics combined with mutations form the basis of the risk classification system, which facilitates the risk stratification for patients. However, up to 50% patients have been diagnosed as intermediate risk AML with a wide range of clinical outcomes. Thus, the identification of vital mechanisms affecting AML management and patient survival may boost the development of AML specific targeted therapies and meticulous risk stratification.

The results from current study indicated that high cGAS and STING expression correlated to inferior survival in AML. In multivariate analysis, only cGAS expression was found to be an independent factor affecting OS and DFS. It was intriguing that the expression level of STING showed impact on OS but not on DFS. Since our result showed cGAS and STING had a strong positive correlation, there might exist an overlapped effect of cGAS and STING expression on the clinical outcomes. Besides, recent work has demonstrated that cGAS and STING may act in an independent way from one another. Upon etoposide-induced DNA damage, STING could be activated independently from the catalytic function of cGAS [23]. The exquisite molecular mechanism defining the acting pattern of cGAS and STING in AML remained undetermined. In this regard, future studies are needed to investigate whether combination of cGAS and STING expression detection is necessary and cGAS gene expression alone can be an indicator for prognosis in AML.

Previous studies indicated the activation of cGAS-STING pathway contributed to cancer suppression by promoting host immuno-surveillance and inducing cellular senescence [24–27]. In line with these findings, cGAS-STING pathway was identified as a prognostic biomarker for improved clinical outcomes in hepatocellular carcinoma and non-small cell lung cancer (NSCLC) [19, 28]. However, other studies revealed that cGAS-STING pathway promoted tumor development and progression in Lewis lung carcinoma, brain and colorectal cancer [14, 15, 29]. One possible mechanism was that chronic stimulation of cGAS-STING pathway might lead to inflammation-driven carcinogenesis [30]. The topic that pro-inflammatory mediators are linked to AML cell growth has gain traction in recent years. Plenty of studies reported that chronic immune-stimulatory or autoimmune disease could be sick factors for developing AML [31, 32]. Here, our results suggested that cGAS-STING pathway also had a potential role in driving malignant programs and suppressing antitumor functions in AML. Whether hyper-activation of cGAS-STING pathway fueled the progression of AML via induction of inflammation requires further exploration. Also, future studies will be required to elucidate mechanism of hyper-activation cGAS-STING signaling in AML.

At present, NRAS/KRAS mutations are widely considered to be associated with poor prognosis in a variety of cancers, including colorectal cancer and serous ovarian cancer [33, 34]. NRAS mutations were identified in 10%–11% of AML, and KRAS mutations in an additional 5% [35, 36], however, how it affects cGAS-STING levels in AML has not been reported. Only one study in KRAS-mutant non-small-cell lung carcinoma lung cancer (NSCLC) may be relevant. In this study, the authors found that NRF2 promotes the transcription and expression of BRCA1 to repair DNA damage, leading to inactivation of the STING pathway [37]. In our study, the frequency of NRAS/KRAS mutation was higher in both cGAS and STING high group. TBK1 is the important downstream effector program of cGAS-STING signaling [7]. Previous work presented that TBK1 supported key, context specific tumorigenic activity in Ras-mutant/mesenchymal NSCLC [38]. The interaction between cGAS-STING pathway including its downstream signaling with NRAS/KRAS mutation on the context of AML would be an interesting area of research. Additionally, we found that elevated expression of cGAS was associated with higher risk stratification and a lower CR rate. Chromosomal instability (CIN) is a hallmark of cancer as well as a primary source of cytosolic dsDNA and it promotes the activation of cGAS-STING [39]. Intriguingly, some scholars found that cGAS could exert the function of maintaining CIN, which potentiated tumor evolution [40, 41]. In our study, AML patients had shown hyper-activation of the cGAS-STING pathway before treatment. The following chemotherapy further induced CIN, which might cooperate with cGAS-STING, leading to further perturbation of this pathway. Yet, the underlying mechanisms that define the effect of cGAS-STING pathway on treatment response in AML patients need further exploration.

In summary, our data revealed a prognosis role of cGAS-STING pathway for clinical outcomes and a positive correlation between cGAS and STING in AML. However, we recognize the limitations to our study, including the limited number of patients enrolled and the lack of relevant mechanism studied. In addition, the cGAS-STING downstream signaling programs including TBK1, IRF3, JAK2/STAT3, NF-κB were not studied in this study. Besides, it is important to note that the dichotomous roles of cGAS-STING in tumor immunity and development are cell and context - dependent [42]. Thus, in future study, a pinpoint cGAS-STING expression state should be better characterized in both immune cells and non-immune cells including tumor cells as well as stromal cell resident in bone marrow environment of AML patients. Despite various approaches for AML investigated, treatment resistance remains a leading cause of AML-related deaths [43]. Based on the results of this study, further studies of novel approaches targeting the cGAS-STING pathway in AML may provide potential for advancing AML therapeutic strategies.

## Data Availability

The original contributions presented in the study are included in the article/supplementary material, further inquiries can be directed to the corresponding authors.

## Footnotes

1
http://gepia.cancer-pku.cn

2
http://tcga-data.nci.nih.gov/tcga/

3
http://www.gtexportal.org/home/index.html

4
http://www.ncbi.nlm.nih.gov/geo
